# Mr. White's Case of Cancerous Hydatids in the Breast

**Published:** 1809-11

**Authors:** W. White

**Affiliations:** Bath


### *To the Editors of the Medical and Physical Journal.*

GENTLEMEN,

MRS. G——, upwards of 70 years of age, of a slender habit of body, and sallow complexion, applied to me in August, 1808, on account of a cancer which she had in her right breast. The mamma was enlarged to a very great size, of an irregular figure, and considerably indurated. The integuments were discoloured; the veins enlarged; there were several soft protuberances, and the skin over them had a shining red appearance, as if ready to break; which event I daily expected for some time. There was also, towards the left side of the breast, a large fungus, which projected considerably beyond the integuments, and had gradually increased since the breast broke in April, 1808. On the right side of the fungus there was a deep cleft, the surrounding edges were hard, and retorted. There was a profuse serous discharge, (extremely offensive to the smell) so much that several thick folded cloths were commonly wet through, although the part was dressed twice a day. Under the breast there were several small, hard lumps, of a livid colour. There was no attachment to the pectoral muscle, nor any enlargement of the axiliary glands. She complained of a pricking pain in the breast, as if a pin or needle was making its way through the skin; the pain however was not so great as might have been expected from the extent of the disease, as she was able to endure it without having recourse to opium. Her strength was declining, and appetite impaired; pulse was quick and feeble; and occasionally she had profuse night perspirations. The following is Mrs. G———'s own history of the disease, previous to her application to me.

"Dear Sir,

"I think it is rather more than eight years since I first discovered a little swelling in the back part of my right breast, which had been attended some time before with a considerable itching, but without any pain. A few months after, I applied to a medical gentleman in Bath, who seemed to think very lightly of the case, and only advised an application of spirit of Mindereri to the affected part. I occasionally took a little bark, and lived more upon a cooling diet than usual. As the swelling seemed to increase a little, I applied again to the same gentleman, but he did not advise any other method, only observed, that he had seen more injury occasioned by the knife and caustic in these cases, than had happened by letting the disease alone. For many years after, the complaint proceeded very slowly; there were also three small knots attending it, but scarcely giving any pain. In November, 1807, as the disease had very much increased, I was induced to go to London, where I consulted several of the most eminent of the Faculty, who differed in their opinion respecting the case, as some of them advised taking off the breast, whilst others were opposed to it, therefore I did not consent to the operation. After this I went into the country a few months, and then returned to London, when one of the surgeons who had seen the breast before, was rather surprized to find it had not broke. As it appeared nothing could be done except removing the breast; in the month of March 1808, I came to Bath, and early in April the breast broke, and soon after had several pieces of black flesh at different times taken out of the wound, which were very offensive to the smell. The complaint became much worse afterwards; general swelling increased, and fungus flesh to a considerable degree, with increase of pain. Towards the end of August I applied to you, since which I have received much benefit, as the application of the powder has reduced the painful fungus flesh, which had had arisen to a vast height, and happily also put a stop to the offensive smell from the wound, and it seemed in a great degree to abate the general pain likewise. 'Tis astonishing how little pain has attended it, not depriving me of one night's rest. I can never be sufficiently thankful to Almighty God for these alleviating mercies.

"I remain, &c.

"G. G."

---

Having read the cases of cancer published by Mr. Carmichael, wherein he had employed the different preparations of iron with so much success, I proposed to Mrs. G——, a trial of the same means; and as a further encouragement, shewed her the cases as detailed by Mr. Carmichael: after which she very readily consented, and the plan was immediately adopted; although, I confess, with very little hope of affording any relief in such a deplorable case.

I directed her to take ten grains of the carbonate of iron twice a day, which was afterwards increased to fifteen grains each dose. The fungus was thickly covered with the phosphate of iron, and also introduced into the cleft, over which was laid some mild ointment, spread upon soft linen. In consequence of this mode of treatment, a daily sloughing took place, and I had the pleasure of seeing, in the course of a few weeks, not only the fungus reduced to a level with the surrounding integuments, but to a considerable depth below. It is also worthy of observation, that there was no longer any fœtor accompanying the discharge; neither did it return, although some time ago, I left off the powder a week; the reason of which was, Mrs. G—— thought the application gave her more pain than formerly; the cause, however, was evidently owing to a large black mass which protruded through the ulcerated surface, for after removing it she was easier. Previous to that period, and likewise since, I have occasionally detached several hydatids; and about three weeks ago, a cluster of them, resembling a small bunch of grapes, appeared on the surface of the ulcer, which I removed with the knife, without exciting pain. The hydatids were so soft as not to bear the pressure of the forceps, being nearly of the consistence of jelly.

The above mentioned favourable appearances were also accompanied with an evident improvement in Mrs. G—'s general health; as she grew stronger, and her appetite for food became much keener than it had been before she was attacked with the complaint. It is now thirteen months since the phosphate of iron was first applied, and I am persuaded the breast is not more than half the size it was at that time: it is also much softer, and the integuments are of a more natural colour, and the surface is become even. The right side of the ulcer, which assumes the hydatid form of the disease, is very soft, and a probe may be passed to a great depth without exciting pain.* The edges of the ulcer on this side have a soft natural feel, but the ulcerated surface on the left side of the breast has more of gristly appearance, and the edges are not so soft as those on the right side. On pressing the under part of the breast towards the left side, a discharge of white matter commonly follows, and where she feels most uneasiness. Some of the lumps which were under the breast have entirely disappeared, and two which remain are become soft, and the skin is less discoloured. The discharge from the breast is still copious. She has no night perspiration.

I am, &c.

W. WHITE.

*Bath, Oct.* 12, 1809.

* On touching, however, a part near the centre of the ulcer (which appears more fibrous than the surrounding surface) exquisite pain is immediately excited.

